# Supplementary material for: LPS-Induced Mortality in Zebrafish: Preliminary Characterisation of Common Fish Pathogens
Source: Microorganisms. 2023 Aug 31;11(9):2205. doi: 10.3390/microorganisms11092205 (PMC10535040; doi:10.3390/microorganisms11092205)
Supplement: Supplementary file 1 [file microorganisms-11-02205-s001.zip › microorganisms-2418495-supplementary.pdf]

SUPPLEMENTARY TABLES

Table S1. *p*-values of pairwise comparisons using Log-rank (Mantel-Cox) test of zebrafish survival curves when subjected to live *A. hydrophila* at the described concentrations (CFU mL^{-1}).

	**Control**	**1e^{9} CFU mL^{-1}**	**5e^{9} CFU mL^{-1}**
**Control**	-	-	-
**1e^{9} CFU mL^{-1}**	>0.9999	-	-
**5e^{9} CFU mL^{-1}**	0.0205	0.0205	-
**1e^{10} CFU mL^{-1}**	<0.0001	<0.0001	0.0006

Table S2. *p*-values of pairwise comparisons using Log-rank (Mantel-Cox) test of zebrafish survival curves when subjected to LPS extracted from *A. hydrophila* (LPS AH) and commercial LPS from *P. aeruginosa* (PA-Com), at the described concentrations (µg mL^{-1}).

	**Control**	**LPS AH 50 µg mL^{-1}**	**LPS AH 100 µg mL^{-1}**	**LPS AH 250 µg mL^{-1}**	**LPS AH 500 µg mL^{-1}**
**Control**	-	-	-	-	-
**LPS AH 50 µg mL^{-1}**	0.2471	-	-	-	-
**LPS AH 100 µg mL^{-1}**	0.2471	>0.9999	-	-	-
**LPS AH 250 µg mL^{-1}**	0.2471	>0.9999	>0.9999	-	-
**LPS AH 500 µg mL^{-1}**	0.0005	0.0005	0.0005	0.0005	-
**LPS PA-Com 45 µg mL^{-1}**	<0.0001	<0.0001	<0.0001	<0.0001	<0.0001

Table S3. *p*-values of pairwise comparisons using Log-rank (Mantel-Cox) test of zebrafish survival curves when subjected to live *V. harveyi* at the described concentrations (CFU mL^{-1}).

	**Control**	**1e^{9} CFU mL^{-1}**	**5e^{9} CFU mL^{-1}**
**Control**	-	-	-
**1e^{9} CFU mL^{-1}**	<0.0001	-	-
**5e^{9} CFU mL^{-1}**	<0.0001	0.1538	-
**1e^{10} CFU mL^{-1}**	0.0012	<0.0001	<0.0001

Table S4. *p*-values of pairwise comparisons using Log-rank (Mantel-Cox) test of zebrafish survival curves when subjected to LPS extracted from *V. harveyi* (LPS VH) commercial LPS from *P. aeruginosa* (PA-Com), at the described concentrations (µg mL^{-1}).

	**Control**	**LPS VH 50 µg mL^{-1}**	**LPS VH 100 µg mL^{-1}**	**LPS VH 250 µg mL^{-1}**	**LPS VH 500 µg mL^{-1}**
**Control**	-	-	-	-	-
**LPS VH 50 µg mL^{-1}**	0.1563	-	-	-	-
**LPS VH 100 µg mL^{-1}**	0.4131	>0.9999	-	-	-
**LPS VH 250 µg mL^{-1}**	0.0062	0.0004	0.0378	-	-
**LPS VH 500 µg mL^{-1}**	<0.0001	<0.0001	<0.0001	<0.0001	-
**LPS PA-Com 45 µg mL^{-1}**	<0.0001	<0.0001	<0.0001	<0.0001	<0.0001

Table S5. *p*-values of pairwise comparisons using Log-rank (Mantel-Cox) test of zebrafish survival curves when subjected to live *Ph. damselae* subps. piscicida at the described concentrations (CFU mL^{-1}).

	**Control**	**1e^7 CFU mL^{-1}**	**2e^7 CFU mL^{-1}**
**Control**	-	-	-
**1e^7 CFU mL^{-1}**	>0.9999	-	-
**2e^7 CFU mL^{-1}**	0.0205	0.0205	-
**5e^7 CFU mL^{-1}**	<0.0001	<0.0001	<0.0001

Table S6. *p*-values of pairwise comparisons using Log-rank (Mantel-Cox) test of zebrafish survival curves when subjected to LPS extracted from *Ph. damselae* subps. piscicida (LPS PDP) commercial LPS from *P. aeruginosa* (PA-Com), at the described concentrations (µg mL^{-1}).

	**Control**	**LPS PDP 100 µg mL^{-1}**	**LPS PDP 500 µg mL^{-1}**
**Control**	-	-	-
**LPS PDP 100 µg mL^{-1}**	>0.9999	-	-
**LPS PDP 500 µg mL^{-1}**	0.3173	0.3173	-
**LPS PA-Com 45 µg mL^{-1}**	<0.0001	<0.0001	<0.0001

Table S7. *p*-values of pairwise comparisons using Log-rank (Mantel-Cox) test of zebrafish survival curves when subjected to LPS extracted from *T. maritimum* (LPS PDP) and LPS commercially available from *P. aeruginosa* (LPS PA Sigma), at the described concentrations (µg mL^{-1}).

	**Control**	**LPS TM 50 µg mL^{-1}**	**LPS TM 100 µg mL^{-1}**	**LPS TM 250 µg mL^{-1}**	**LPS TM 500 µg mL^{-1}**
**Control**	-	-	-	-	-
**LPS TM 50 µg mL^{-1}**	0.3173	-	-	-	-
**LPS TM 100 µg mL^{-1}**	0.6097	0.1573	-	-	-
**LPS TM 250 µg mL^{-1}**	0.9961	0.3173	0.6097	-	-
**LPS TM 500 µg mL^{-1}**	<0.0001	<0.0001	<0.0001	<0.0001	-
**LPS PA-Com 45 µg mL^{-1}**	<0.0001	<0.0001	<0.0001	<0.0001	<0.0001

Table S8. *p*-values of pairwise comparisons using Log-rank (Mantel-Cox) test of zebrafish survival curves when subjected to commercial LPS from *P. aeruginosa* (PA-Com), and LPS extracted from *P. aeruginosa* (LPS PA Ext) at the described concentrations (µg mL^{-1}).

	**Control**	**LPS PA-Com 50 µg mL^{-1}**	**LPS PA-Com 100 µg mL^{-1}**	**LPS PA Ext 50 µg mL^{-1}**	**LPS PA Ext 100 µg mL^{-1}**
**Control**	-	-	-	-	-
**LPS PA-Com 50 µg mL^{-1}**	<0.0001	-	-	-	-
**LPS PA-Com 100 µg mL^{-1}**	<0.0001	<0.0001	-	-	-
**LPS PA Ext 50 µg mL^{-1}**	>0.9999	<0.0001	<0.0001	-	-
**LPS PA Ext 100 µg mL^{-1}**	0.1544	<0.0001	<0.0001	0.1544	-
**LPS PA Ext 250 µg mL^{-1}**	0.3173	<0.0001	<0.0001	0.3173	0.5729
